# Mapping Isoflavone QTL with Main, Epistatic and QTL × Environment Effects in Recombinant Inbred Lines of Soybean

**DOI:** 10.1371/journal.pone.0118447

**Published:** 2015-03-04

**Authors:** Yan Wang, Yingpeng Han, Xue Zhao, Yongguang Li, Weili Teng, Dongmei Li, Yong Zhan, Wenbin Li

**Affiliations:** 1 Key Laboratory of Soybean Biology in Chinese Ministry of Education, Northeast Agricultural University, Harbin, Heilongjiang Province, People’s Republic of China; Key Laboratory of Soybean Biology and Breeding/Genetics of Chinese Agriculture Ministry, Northeast Agricultural University, Harbin, Heilongjiang Province, People’s Republic of China; 2 Agricultural Science Academy of Shi He Zi, Xinjiang Province, People’s Republic of China; College of Agricultural Sciences, UNITED STATES

## Abstract

Soybean (*Glycine max* (L.) Merr.) isoflavone is important for human health and plant defense system. To identify novel quantitative trait loci (QTL) and epistatic QTL underlying isoflavone content in soybean, F_5:6_, F_5:7_ and F_5:8_ populations of 130 recombinant inbred (RI) lines, derived from the cross of soybean cultivar ‘Zhong Dou 27′ (high isoflavone) and ‘Jiu Nong 20′ (low isoflavone), were analyzed with 95 new SSR markers. A new linkage map including 194 SSR markers and covering 2,312 cM with mean distance of about 12 cM between markers was constructed. Thirty four QTL for both individual and total seed isoflavone contents of soybean were identified. Six, seven, ten and eleven QTL were associated with daidzein (DZ), glycitein (GC), genistein (GT) and total isoflavone (TI), respectively. Of them 23 QTL were newly identified. The qTIF_1 between Satt423 and Satt569 shared the same marker Satt569 with qDZF_2, qGTF_1 and qTIF_2. The qGTD2_1 between Satt186 and Satt226 was detected in four environments and explained 3.41%-10.98% of the phenotypic variation. The qGTA2_1, overlapped with qGCA2_1 and detected in four environments, was close to the previously identified major QTL for GT, which were responsible for large *a* effects. QTL (qDZF_2, qGTF_1 and qTIF_2) between Satt144-Satt569 were either clustered or pleiotropic. The qGCM_1, qGTM_1 and qTIM_1 between Satt540-Sat_244 explained 2.02%–9.12% of the phenotypic variation over six environments. Moreover, the qGCE_1 overlapped with qGTE_1 and qTIE_1, the qTIH_2 overlapped with qGTH_1, qGCI_1 overlapped with qDZI_1, qTIL_1 overlapped with qGTL_1, and qTIO_1 overlapped with qGTO_1. In this study, some of unstable QTL were detected in different environments, which were due to weak expression of QTL, QTL by environment interaction in the opposite direction to *a* effects, and/or epistasis. The markers identified in multi-environments in this study could be applied in the selection of soybean cultivars for higher isoflavone content and in the map-based gene cloning.

## Introduction

Soy food is considered as the functional food because it contains health beneficial molecules such as isoflavone [1–2]. Human nutrition studies have shown that isoflavone plays an important role in preventing a number of chronic diseases [3–5]. Meanwhile, isoflavone has been approved to be a critical factor in defending against plant pests [6–9], in promoting nodulation by rhizobia [10–12], and in changing or adjusting the microorganisms found around plant roots [13]. Soybean isoflavone include daidzein (DZ), genistein (GT), glycitein (GC), daidzin, genistin, glycitin, 6-o-acetyldaidzin, 6-o-acetylgenistin, 6-o-acetylglycitin, 6-o-malonyldaidzin, 6-o-malonylgenistin and 6-o-malonylglycitin. Among them, DZ, GT and GC are the major bioactive components in human nutrition. Soybean isoflavone concentrations are heritable but behave as complex quantitative traits [14–18] regulated by multiple environmental and genetic factors [1, 19–23]. Hence, selection for soybean cultivars with varied seed isoflavone contents required evaluation in multiple environments over several years, which is expensive, time-consuming and labor intensive.

Traditional methods of genetic improvement of quantitative traits were mainly dependent on phenotypic information [24], which was readily affected by environmental factors. Molecular markers offered a faster and more accurate approach for breeding, because selection could be based on genotype rather than solely on phenotype. The use of molecular markers in the selection of important agronomic traits, or marker-assisted selection (MAS) could improve the efficiency of traditional plant breeding [25–27]. However, the use of MAS requires knowledge of reliable marker-trait associations that are relatively stable over multiple environments, because constant QTL over multiple environments might contribute to a consistent phenotype beneath changing conditions.

An individual QTL is described as ‘major’ or ‘minor’ is based on the proportion of the phenotypic variation explained by a QTL (based on the R^2^ value). Major QTL will account for a relatively large amount of R^2^ value (R^2^ > 10%), while minor QTL will usually account for a relatively small amount of R^2^ value (R^2^ < 10%) [28]. Sometimes, major QTL may refer to QTL that is stable across environments whereas minor QTL may refer to QTL that is environmentally sensitive [28]. Because soybean seed isoflavone accumulation is mainly dominated by minor-effect QTL that are often influenced by environment, discovering a stable QTL has been hindered. Therefore, it is imperative to identify some stable loci associated with isoflavone content in different environments.

To boost QTL detection, appropriate crosses need to be selected to generate sufficient genetic variations in genomic level [29]. Although a linkage map is not strictly required for MAS, a dense marker genetic map greatly facilitates strong marker—gene correlations by permitting the utilization of improved QTL mapping approaches [30].

Over fifty QTLs underlying individual and/or individual and total isoflavone content have been reported [14–17, 31–37]. Among them, two QTL with main effects, located in Gm05 (soybean chromosome 5, LGA1) and Gm08 (LGA2), respectively, consistently affected isoflavone content across multi-environments [30, 35]. Striking eQTL hotspots associated with soybean seed-specific expression on Gm 20, 7, and 13 were discovered by Bolon et al. [38]. Wang et al. [39] identified seven candidate genes on Gm13 (LG F) and assumed Gm13 could be a hotspot of gene cluster that regulated seed isoflavone content.

Epitasis is also thought to contribute to isoflavone variation [17]. The result of some studies indicated that significant QTL epistatic interactions influenced isoflavone content in soybean seed [17, 32]. Hence, the isoflavone content in soybean seed is considered as complex quantitative trait because their levels are highly variable and regulated by multiple genetic and environmental factors [1, 19–23].

Zeng et al. [31] identified fifteen QTL underlying seed isoflavone contents of soybean based on a RI line population derived from a cross between ‘Zhong Dou 27′ and ‘Jiu Nong 20′ through a genetic linkage map including 99 SSR markers. In the present work, 95 additional SSR markers were added to the map of Zeng et al. [31] to identify novel QTL with main, epistatic or QTL × environment effects that associated with seed isoflavone contents of soybean.

## Materials and Methods

### Plant materials and growing conditions

The mapping populations of 130 F_5:6,_ F_5:7,_ F_5:8_ recombinant inbred lines (RIL) that were advanced by single-seed-descent from the cross between ‘Zhong Dou 27′ (developed by the Chinese Academy of Agricultural Sciences, Beijing, China) and ‘Jiu Nong 20′ (developed by Jilin Academy of Agricultural Sciences, Jilin, China). ‘Zhong Dou 27′ contained high individual and total isoflavone (TI) contents (daidzein, DZ, 1,865 μg/g; genistein, GT, 1,614 μg/g; glycitein, GC, 311 μg/g and total isoflavone, TI, 3,791 μg/g), whereas ‘Jiu Nong 20′ had low individual and TI contents (DZ, 844 μg/g; GT, 1,046 μg/g; GC, 193 μg/g and TI, 2,061 μg/g). The 130 RILs of F_5:6_, F_5:7,_ F_5:8_ and parents were planted at Harbin, Hulan and Suihua in 2005, 2006 and 2007, respectively [31]. Randomized complete block designs were used for all experiments with rows 3 m long, 0.65 m apart, and a space of 6 cm between plants.

### The screening of simple sequence repeats (SSR) markers

Total DNA of the parents and each RI line were isolated from dried leaf tissues by CTAB method [40]. A total of 500 additional SSR markers, covering the whole genome of soybean (available at http://www.soybease.org), were used to detect polymorphisms. The PCR reactions were 94°C for 2 min, followed by 35 cycles of 30 s at 94°C, 30 s at 52°C, 30 s at 72°C and 5 min at 72°C after the last cycle. The amplified PCR products were mixed with loading buffer and denatured for 5 min at 94°C and then kept on ice for 5 min. The denatured PCR products were separated on 6% (w/v) denaturing polyacrylamide gel and visualized by silver staining [41]. The polymorphic SSR markers were integrated into the map constructed by Zeng et al. [31].

### QTL analyses

Polymorphic markers were identified and mapped on the 20 linkage groups by Mapmaker 3.0b with the Kosambi mapping function [42]. WinQTLCart2.1 [43] was used to detect QTL between marker intervals by 1,000 permutations at significance (*P≤ 0*.*05*). The genetic linkage map was constructed using Mapchart 2.1 [44]. QTL genetic effects including additive, additive × additive epistatic effects and their environmental interaction effects were analyzed according to the method of Wang et al. [45]. The nomenclature of the QTL included four parts following the recommendations of the Soybean Germplasm Coordination Committee. For example, qDZI_1, q, DZ, I and 1 represent QTL, trait (daidzein, DZ), linkage group name and QTL order in the linkage group, respectively.

GGE Bioplot methodology [46] was employed to analyze the interaction between QTL and environments, based on the formula: T_ij_ − T_j_ / S_j_ = λ_1_ζ_i1_τ_j1_ + λ_2_ζ_i2_τ_j2_ + ε_ij_, where T_ij_ was the mean value of QTL i for environment j; T_j_ was the mean value of environment j over all QTL, S_j_ was the standard deviation of environment j among QTL mean; ζ_i1_ and ζ_i2_ were the PC1 (first principle component) and PC2 (second principle component) scores respectively, for QTL mean i; τ_j1_ and τ_j2_ were the PC1 and PC2 scores respectively, for environment j; and ε_ij_ was the residual of the model associated with QTL i, challenged with environment j.

## Results

### Linkage analyses

In this study, a total of 500 new SSR markers were used to detect polymorphisms between the two parents, 95 of which were integrated into the linkage map constructed by Zeng et al [31]. This linkage map included 194 SSR markers and covered 2,312.16 cM with mean distance of 11.92 cM between markers.

### Identification of QTL for total and individual isoflavone contents

**Fig 1:**
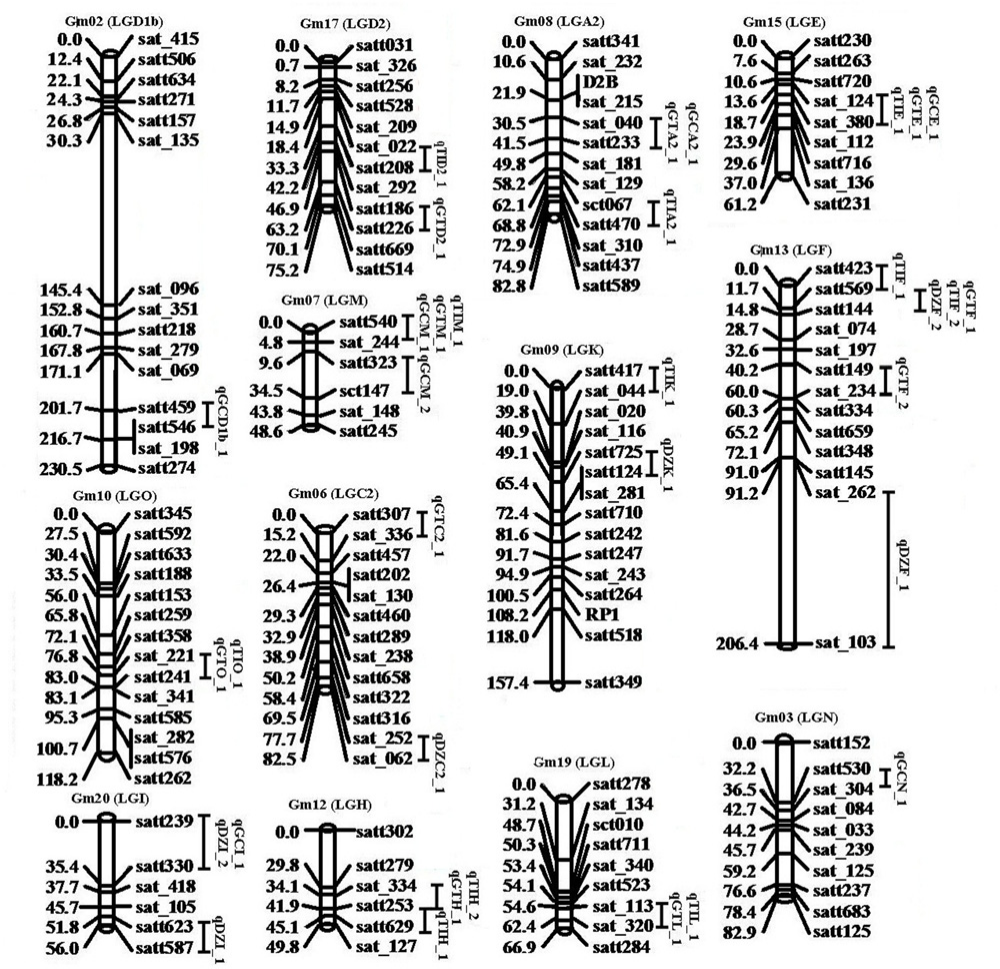
Summary of QTL locations detected in the soybean genome. QTL represented by bars were shown on the left of the linkage groups, close to their corresponding markers. The lengths of the bars were proportional to the confidence intervals of the corresponding QTL in which the inner line indicates the position of maximum LOD score.

In this study, the identification of QTL was based on multi-environmental phenotypic data of Zeng et al. [31]. Thirty four QTL underlying individual and total isoflavone contents were identified on thirteen LGs in seven environments over three years (Table 1, Fig. 1). Among them, six, seven, ten and eleven QTL were associated with DZ, GC, GT and TI, respectively.

**Table 1:** QTLs for individual and total isoflavone content.

Traits^a^	QTL^b^	Gm (LG)	Marker	Marker interval	Position ^c^	Environment ^g^	LOD score	R^2^(%) ^d^
**DZ**	^e^qDZC2_1	06(C2)	Sat_252	Sat_252-Sat_062	77.67	E1	2.09	5.60
						E3	2.45	6.41
	qDZF_1	13(F)	Sat_103	Sat_103-Sat_262	188.34	E2	2.00	10.57
	qDZI_2	20(I)	Satt330	Satt330-Satt239	32.87	E1	3.49	7.82
						E2	3.74	7.62
						E7	2.24	9.93
	^f^qDZF_2	13(F)	Satt144	Satt144-Satt569	14.15	E2	4.15	8.01
						E3	4.68	9.00
						E4	2.76	2.98
						E5	2.17	3.01
						E6	4.73	10.32
						E7	2.78	8.75
	qDZI_1	20(I)	Satt587	Satt587-Satt623	55.38	E3	4.90	9.23
						E5	2.80	6.52
						E6	2.04	8.78
						E7	2.38	5.03
	qDZK_1	09(K)	Satt124	Satt124-Satt725	60.35	E2	2.46	1.64
						E6	3.68	4.82
						E7	2.05	6.43
**GC**	^e^qGCA2_1	08(A2)	Sat_040	Sat_040-Satt233	38.46	E3	2.65	6.01
	qGCD1b_1	02(Dlb)	Satt546	Satt546-Sat_459	215.67	E2	2.38	3.12
						E5	2.21	4.17
	qGCE_1	15(E)	Sat_124	Sat_124-Sat_380	16.21	E1	2.34	2.33
						E4	2.02	8.49
						E7	2.03	1.43
	qGCN_1	03(N)	Satt530	Satt530-Sat_304	34.57	E4	2.41	5.21
	qGCM_2	07(M)	Sct_147	Sct_147-Satt323	30.66	E1	3.20	7.01
						E4	4.44	2.87
	^f^ qGCI_1	20(I)	Satt330	Satt330-Satt239	32.92	E1	3.89	5.01
						E3	2.33	6.67
						E4	2.43	5.32
						E5	2.26	2.16
	qGCM_1	07(M)	Satt540	Satt540-Sat_244	2.37	E2	2.77	2.02
						E4	2.11	6.42
						E5	3.48	4.45
						E7	3.56	4.21
**GT**	^e^ qGTC2_1	06(C2)	Satt307	Satt307-Sat_336	5.86	E6	2.48	15.16
	qGTD2_1	17(D2)	Satt186	Satt186-Satt226	50.81	E1	2.00	3.41
						E2	2.36	5.23
						E3	5.76	10.98
						E5	3.09	8.23
	qGTE_1	15(E)	Sat_124	Sat_124-Sat_380	15.61	E7	2.58	5.37
	qGTF_2	13(F)	Satt149	Satt149-Sat_234	41.23	E1	2.00	1.56
						E3	2.49	4.17
						E7	4.03	5.47
	qGTH_1	12(H)	Sat_334	Sat_334-Satt253	37.65	E4	2.97	17.05
	qGTL_1	19(L)	Sat_113	Sat_113-Sat_320	58.63	E1	3.00	12.01
						E3	2.15	4.97
						E4	3.64	4.74
	qGTO_1	10(O)	Sat_221	Sat_221-Satt241	78.51	E1	3.21	5.01
						E6	3.07	5.83
	^f^ qGTF_1	13(F)	Satt144	Satt144-Satt569	13.46	E1	2.12	2.01
						E2	2.74	7.96
						E4	2.20	6.40
						E5	5.68	17.03
						E6	2.25	5.04
						E7	2.37	6.01
	qGTM_1	07(M)	Satt540	Satt540-Satt244	1.66	E2	2.36	2.67
						E3	3.56	3.73
						E6	3.77	7.98
	qGTA2_1	08(A2)	Sat_040	Sat_040-Satt233	35.7	E2	3.45	7.01
						E3	2.37	3.51
						E5	2.42	11.58
						E7	2.01	4.01
**TI**	^e^ qTIA2_1	08(A2)	Sct_067	Sct_067-Satt470	65.83	E7	2.16	1.39
	qTIE_1	15(E)	Sat_124	Sat_124-Sat_380	15.64	E5	2.35	1.20
						E6	3.77	3.41
	qTIF_1	13(F)	Satt423	Satt423-Satt569	6.01	E6	4.59	3.21
						E7	2.15	4.20
	qTIH_1	12(H)	Satt253	Satt253-Satt629	43.37	E3	2.33	4.74
						E4	2.38	5.95
	qTIH_2	12(H)	Satt253	Satt253-Satt334	39	E2	3.26	7.03
	qTIK_1	09(K)	Satt417	Satt417-Sat_044	2.16	E7	2.15	11.62
	qTIL_1	19(L)	Sat_113	Sat_113-Sat_320	58.11	E1	2.00	1.50
						E2	2.33	4.03
						E5	5.67	6.19
						E6	2.47	3.13
	qTID2_1	17(D2)	Sat_022	Sat_022-Satt208	21.53	E1	3.12	1.46
						E2	2.30	2.11
						E7	2.28	6.71
	^f^ qTIM_1	07(M)	Satt540	Satt540-Sat_244	2.37	E4	4.32	7.34
						E5	2.65	4.17
						E6	4.24	9.12
	qTIO_1	10(O)	Satt241	Satt241-Sat_221	80.48	E2	2.24	5.62
						E3	2.42	1.75
	qTIF_2	13(F)	Satt144	Satt144-Satt569	13.46	E2	3.17	2.03
						E4	2.89	2.95
						E5	2.19	1.87
						E6	2.77	7.96
						E7	2.45	3.87

^a^ DZ: Daidzein; GC:Glycitein; GT: Genistein; TI: Total isoflavone

^b^ The nomenclature of the QTL included four parts: QTL, trait, linkage group name and QTL order in the linkage group, respectively

^c^ Position from the left marker of the interval on each linkage group

^d^ Proportion of phenotypic variance (R^2^) explained by a QTL

^e^ Additional QTL for individual and total isoflavone content

^f^ QTL in accordance with Zeng et al. [30]

^g^ E1: at Harbin in 2005, E2: at Harbin in 2006, E3: at Hulan in 2006, E4:at Suihua in 2006, E5: at Harbin in 2007, E6: at Hulan in 2007, E7: at Suihua in 2007

Three QTL (qDZF_2, qGTF_1, qTIF_2) associated with DZ, GT and TI were located on Gm13 (LG F) between Satt144-Satt569. They explained 1.87%–17.03% of the phenotypic variation in seven environments over three years. Another three QTL associated with GC, GT and TI were located on GM07 (LG M) between Satt540-Sat_244. They explained 2.02%–9.12% of the phenotypic variation across seven environments.

Among the 34 QTL, 23 QTL were newly identified. Most of them were detected in one or two environments and explained 1.43%-17.05% of the phenotypic variation. The qTIF_1 in the interval between Satt423 and Satt569 shared the same marker Satt569 with qDZF_2, qGTF_1 and qTIF_2. The qGTD2_1 between Satt186 and Satt226 was detected in four environments and explained 3.41%–10.98% of the phenotypic variation. The qGTA2_1 was detected in four environments, which shared the same marker interval of Sat_040-Satt233 with qGCA2_1.

Interestingly, six pairs of QTL overlapped with each other and shared the same marker interval (Table 1, Fig. 1). For example, the qGCE_1 overlapped with qGTE_1 and qTIE_1 in the interval between Sat_112 and Sat_380, the qGTO_1 overlapped with qTIO_1 in the interval between Sat_221 and Satt241, the qDZI_2 overlapped with qGCI_1 in the interval between Satt330 and Satt239, the qGCA2_1 overlapped with GTA2_1 in the interval between Sat_040 and Satt233, the qGTH_1 overlapped with qTIH_2 in the interval between Sat_334 and Satt253 and the qGTL_1 overlapped with qTIL_1 in the interval between Sat_113 and Sat_320.

### QTL × environment interaction

Six, seven, ten and eleven QTL associated with DZ, GC, GT and TI respectively, had additive main effect (*a*) and/or additive × environment interaction effect (*ae*) at certain environments (Table 2). Two QTL (qDZI_2, qDZF_2) associated with DZ, two QTL (qGCE_1, qGCM_1) associated with GC, four QTL (qGTD2_1, qGTF_1, qGTM_1, qGTA2_1) associated with GT and four QTL (qTIE_1, qTIL_1, qTIM_1, qTIF_2) associated with TI, contributed to the allele that increased individual and total isoflavone through significant *a* effects. Three QTL (qDZC2_1, qDZI_1, qDZK_1) associated with DZ, one QTL (qGCI_1) associated with GC, two QTL (qGTL_1, qGTF_2) associated with GT and three QTL (qTIA2_1, qTID2_1, qTIO_1) associated with TI, contributed to the allele that decreased individual and total isoflavone through significant *a* effects, respectively.

**Table 2:** Additive and additive × environment interaction effect of QTL associated with individual and total isoflavone at RIL population.

Trait^a^	QTL^b^	Gm (LG)	Marker	Marker interval	*a* ^c^	*a*×E1^d^ ^,^ ^e^	*a*×E2	*a*×E3	*a*×E4	*a*×E5	*a*×E6	*a*×E7
**DZ**	qDZC2_1	06(C2)	Sat_252	Sat_252-Sat_062	-0.27*	0.23*		-0.19*				
	qDZF_1	13(F)	Sat_103	Sat_103-Sat_262			-0.10*					
	qDZI_2	20(I)	Satt330	Satt330-Satt239	1.00**	0.30*	0.70**					-0.98**
	qDZF_2	13(F)	Satt144	Satt144-Satt569	1.01**		-0.61**	0.21*	-0.20*	0.59**	-0.92**	0.90**
	qDZI_1	20(I)	Satt587	Satt587-Satt623	-0.32*			0.74**	0.21*		-0.19*	-0.78**
	qDZK_1	09(K)	Satt124	Satt124-Satt725	-0.17*		0.34*				-0.65**	0.30*
**GC**	qGCA2_1	08(A2)	Sat_040	Sat_040-Satt233				-0.08**				
	qGCD1b_1	02(Dlb)	Satt546	Satt546-Sat_459		-0.40*	0.67**			-0.26*		
	qGCE_1	15(E)	Sat_124	Sat_124-Sat_380	1.00**	0.15*			-0.96**			0.79**
	qGCN_1	03(N)	Satt530	Satt530-Sat_304					0.12*			
	qGCM_2	07(M)	Sct_147	Sct_147-Satt323		0.57**			-0.54**			
	qGCI_1	20(I)	Satt330	Satt330-Satt239	-0.24*	0.97**		0.86**	-1.24**	-0.58*		
	qGCM_1	07(M)	Satt540	Satt540-Sat_244	0.74**		0.60**		-0.59**	-0.98**		1.00**
**GT**	qGTC2_1	06(C2)	Satt307	Satt307-Sat_336			0.12*				-0.13*	
	qGTD2_1	17(D2)	Satt186	Satt186-Satt226	0.98**	-0.98*		0.14*		0.71**		
	qGTE_1	15(E)	Sat_124	Sat_124-Sat_380								0.09*
	qGTF_2	13(F)	Satt149	Satt149-Sat_234	-0.34*	0.18*		-0.09*				-0.09*
	qGTH_1	12(H)	Sat_334	Sat_334-Satt253					-0.10*			
	qGTL_1	19(L)	Sat_113	Sat_113-Sat_320	-0.13*	-0.36*		0.20*	0.15*			
	qGTO_1	10(O)	Sat_221	Sat_221-Satt241		-0.18*					0.17*	
	qGTF_1	13(F)	Satt144	Satt144-Satt569	1.00**	0.98*	-0.84**		-0.12*	-1.00**	0.53**	0.49**
	qGTM_1	07(M)	Satt540	Satt540-Satt244	0.56**		-1	0.83**			0.18*	
	qGTA2_1	08(A2)	Sat_040	Sat_040-Satt233	0.67**		0.87*	-0.42*		-1.00**		0.57**
**TI**	qTIA2_1	08(A2)	Sct_067	Sct_067-Satt470	-0.12*				-0.32*	0.30*		-0.13*
	qTIE_1	15(E)	Sat_124	Sat_124-Sat_380	0.39*					0.21*	0.19*	
	qTIF_1	13(F)	Satt423	Satt423-Satt569							1.08**	-1.00**
	qTIH_1	12(H)	Satt253	Satt253-Satt629				0.67**	-0.68**			
	qTIH_2	12(H)	Satt253	Satt253-Satt334			0.07*					
	qTIK_1	09(K)	Satt417	Satt417-Sat_044								-0.14*
	qTIL_1	19(L)	Sat_113	Sat_113-Sat_320	0.78**	0.18*	-0.21*			1.00*	-0.97**	
	qTID2_1	17(D2)	Sat_022	Sat_022-Satt208	-0.34*	0.58**	-0.23*					-0.36*
	qTIM_1	07(M)	Satt540	Satt540-Sat_244	0.54**				0.12*	0.67**	-0.80**	
	qTIO_1	10(O)	Satt241	Satt241-Sat_221	-0.19*		-0.76**	0.78**				
	qTIF_2	13(F)	Satt144	Satt144-Satt569	1.21**		0.31*		-0.55*	0.26*	0.76*	-0.71*

*Significant at P = 0.05,

**Significant at P = 0.01, respectively.

^a^ DZ: Daidzein; GC:Glycitein; GT: Genistein; TI: Total isoflavone.

^b^ The nomenclature of the QTL included four parts: QTL, trait, linkage group name and QTL order in the linkage group, respectively.

^c^ *a*: additive effect.

^d^ *a*×E: additive × environment effect.

^e^ E1: at Harbin in 2005, E2: at Harbin in 2006, E3: at Hulan in 2006, E4: at Suihua in 2006, E5: at Harbin in 2007, E6: at Hulan in 2007, E7: at Suihua in 2007.

The impact of QTL *ae* effects was different across seven environments and three years. For example, the qDZF_2 increased DZ at Hulan in 2006, at Harbin in 2007 and at Suihua in 2007, but decreased DZ at Harbin in 2006, at Suihua in 2006 and at Hulan in 2007. One QTL (qDZF_1) associated with DZ, four QTL (qGCA2_1, qGCD1b_1, qGCN_1, qGCM_2) associated with GC, four QTL (qGTC2_1, qGTE_1, qGTH_1, qGTO_1) associated with GC and four QTL (qTIF_1, qTIH_1, qTIH_2, qTIK_1) associated with TI, had only significant *ae* effects rather than significant *a* effects. Other QTL in seven environments had both significant *a* effects and significant *ae* effects.

### Epistatic analyses of QTL across multi-environments

Six, seven, six and nine epistatic pairwise QTL were associated with DZ, GC, GT and TI content, respectively, in different environments (Table 3). Of them, three, one, three and three epistatic pairs of QTL positively increased DZ, GC, GT and TI content through significant *aa* effects in different environments, respectively. One, one, one and two epistatic pairs of QTL decreased DZ, GC, GT and TI content, respectively, through significant *aa* effects in different environments (Table 3).

**Table 3:** Additive × additive epistatic effect and their environmental interaction effect of QTL associated with individual and total isoflavone at RIL population.

Traits^a^	QTLi^b^	Gm (LG)	Marker	Marker interval	QTLJ^b^	Gm (LG)	Marker	Marker interval	*aa* ^c^	*aa*×E1^d^ ^,^ ^e^	*aa*×E2	*aa*×E3	*aa*×E4	*aa*×E5	*aa*×E6	*aa*×E7
**DZ**	qDZC2_1	06(C2)	Sat_252	Sat_252-Sat_062	qDZI_2	20(I)	Satt330	Satt330-Satt239	-0.29*							
					qDZF_2	13(F)	Sat_103	Sat_103-Sat_262			0.98**			0.17*		-0.92**
	qDZI_2	20(I)	Satt330	Satt330-Satt239	qDZF_2	13(F)	Satt144	Satt144-Satt569	0.98**	0.88**	0.23*	-0.19*		-0.32*		
					qDZI_1	20(I)	Satt587	Satt587-Satt623	0.34*							-0.56**
					qDZK_1	09(K)	Satt124	Satt124-Satt725							0.45*	-0.31*
	qDZF_2	13(F)	Satt144	Satt144-Satt569	qDZI_1	20(I)	Satt587	Satt587-Satt623	1.00**			0.98**	-1.01**	0.41*	-0.31*	0.17*
**GC**	qGCD1b_1	02(Dlb)	Satt546	Satt546-Sat_459	qGCE_1	15(E)	Sat_124	Sat_124-Sat_380			-0.21*					
					qGCI_1	20(I)	Satt330	Satt330-Satt239						0.33*		
	qGCE_1	15(E)	Sat_124	Sat_124-Sat_380	qGCN_1	03(N)	Satt530	Satt530-Sat_304		-0.54**						1.00**
					qGCM_1	07(M)	Satt540	Satt540-Sat_244	0.34*	-0.28*			-0.17*			
	qGCN_1	03(N)	Satt530	Satt530-Sat_304	qGCI_1	20(I)	Satt330	Satt330-Satt239					0.78**			
	qGCM_2	07(M)	Sct_147	Sct_147-Satt323	qGCI_1	20(I)	Satt330	Satt330-Satt239		0.98**						
	qGCI_1	20(I)	Satt330	Satt330-Satt239	qGCM_1	07(M)	Satt540	Satt540-Sat_244	-0.12*			-0.68**	-0.99**	0.25*		
**GT**	qGTD2_1	17(D2)	Satt186	Satt186-Satt226	qGTL_1	19(L)	Sat_113	Sat_113-Sat_320	-0.17*	0.23*	-0.67**	0.98**		-0.12*		
					qGTF_1	13(F)	Satt144	Satt144-Satt569	0.78**	-0.28*	0.43*	-0.12*		0.67**		
	qGTF_2	13(F)	Satt149	Satt149-Sat_234	qGTM_1	07(M)	Satt540	Satt540-Satt244		0.18*		0.09*				
	qGTO_1	10(O)	Sat_221	Sat_221-Satt241	qGTA2_1	08(A2)	Sat_040	Sat_040-Satt233		0.09*						
																
	qGTF_1	13(F)	Satt144	Satt144-Satt569	qGTM_1	07(M)	Satt540	Satt540-Satt244	0.54**	0.12*	0.48**			0.99**	-0.16*	
					qGTA2_1	08(A2)	Sat_040	Sat_040-Satt233	0.23*	0.30*			0.17*	-0.25*		
**TI**	qTIA2_1	08(A2)	Sct_067	Sct_067-Satt470	qTIE_1	15(E)	Sat_124	Sat_124-Sat_380	-0.23*					-0.18*		0.65**
					qTIL_1	19(L)	Sat_113	Sat_113-Sat_320	0.67**	-0.45**	0.20*			1.00**		
					qTID2_1	17(D2)	Sat_022	Sat_022-Satt208		0.11*						
	qTIE_1	15(E)	Sat_124	Sat_124-Sat_380	qTIL_1	19(L)	Sat_113	Sat_113-Sat_320						-0.27*		0.18*
	qTIH_1	12(H)	Satt253	Satt253-Satt629	qTIO_1	10(O)	Satt241	Satt241-Sat_221				-0.09*				
					qTIF_2	13(F)	Satt144	Satt144-Satt569					0.18*			
	qTIL_1	19(L)	Sat_113	Sat_113-Sat_320	qTID2_1	17(D2)	Sat_022	Sat_022-Satt208	-0.09*	0.07*	0.34*				0.31*	
					qTIO_1	10(O)	Satt241	Satt241-Sat_221	0.18*							
	qTIM_1	07(M)	Satt540	Satt540-Sat_244	qTIO_1	10(O)	Satt241	Satt241-Sat_221	0.17*				0.12*	0.06*	-0.76*	

*Significant at P = 0.05,

**Significant at P = 0.01, respectively.

^a^ DZ: Daidzein; GC:Glycitein; GT: Genistein; TI: Total isoflavone.

^b^ The nomenclature of the QTL included four parts: QTL, trait, linkage group name and QTL order in the linkage group, respectively.

^c^ *aa*: additive × additive effect.

^d^ *aa*×E: *aa* × environment effect.

^e^ E1: at Harbin in 2005, E2: at Harbin in 2006, E3: at Hulan in 2006, E4:at Suihua in 2006, E5: at Harbin in 2007, E6: at Hulan in 2007, E7: at Suihua in 2007.

The epistasis × environment interaction effect (*aae*) was an important component of QTL × environment (QE) interaction effects. One QTL and one pairs of QTL were found with only epistatic effects (*aa*), which was associated with DZ and TI content. Two, four, two and four pairs of QTL were found with only epistatic effects (*aa*), which was associated with DZ, GC, GT and TI content, respectively.

### Stability evaluation of QTL associated with individual and total isoflavone contents across mutli-environments

**Fig 2:**
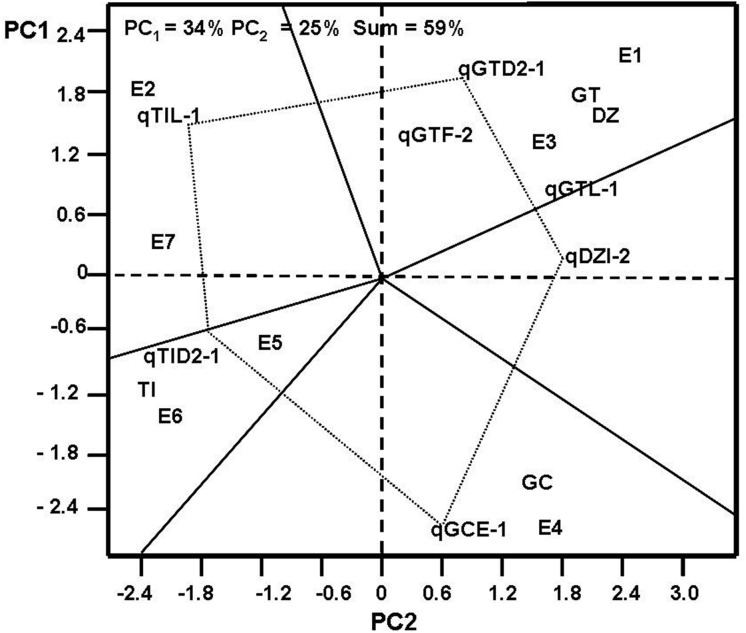
GT biplot analysis for the relatedness of QTL and environment. PC1: first principle component, PC2: second principle component.

GGE Biplot analysis [46] of seven novel main QTL for individual and total isoflavone contents against seven environments showed that these QTL explained 59% of the total variation of seed isoflavone (Fig. 2). The performance of QTL at each environment was evaluated. When QTL (qTIL_1, qGTD2_1, qDZI_2, qGCE_1 and qTID2_1) were set as the corner QTL, seven different environments fell in the sector in which the QTL qGTD2_1 was the best QTL for two environments (at Harbin in 2005 and at Hulan in 2006, Fig. 2). The qTIL_1 was the best QTL at Harbin in 2006 and at Suihua in 2007, and the qTID2_1 was the best one at Hulan in 2007.

## Discussion

Soybean isoflavones have multiple uses in food, medicine, cosmetics and animal husbandry [47]. Improving seed isoflavone content, therefore, should be appeared to be a useful target of soybean breeding. MAS based on genotype selection rather than solely on phenotype provided an outstanding perspective in soybean breeding [30].

‘Zhong Dou 27′ was proved to have high-isoflavone content (3,791 μg/g isoflavone in seed) among seven environments in our previous report [31]. Meng et al. [34] identified two QTL resisting to soybean aphid through isoflavone-mediated antibiosis in soybean cultivar ‘Zhong Dou 27′. These two QTL were significantly associated with high isoflavone content with positive effects derived from ‘Zhong Dou 27′, providing potential for MAS to improve the resistance of cultivar to aphid along with the increase of isoflavone content. This germplasm should be given more attention to reveal the underlying genetic mechanism. In our previous study, a numbers of QTL associated with seed isoflavone were identified in ‘Zhong Dou 27′ using 99 SSR markers. In the present study, additional ninety-five SSR markers were added to the existed linkage map of Zeng et al. [31]. The low level of phenotypic variation evaluated for these QTL in this study (<10%) was indicative of the quantitative nature of individual and total isoflavone, which was similar to the other studies [14–17, 31–36].

qDZF_2, qGTF_1 and qTIF_2 between Satt144-Satt569, and qGCM_1, qGTM_1 and qTIM_1 between Satt540-Sat_244, were identified in multiple environments (Fig. 1). These QTL detected by Satt540 and QTL detected by Satt144 on LG M and on LG F in this study were the same or similar to that of our previous studies [31–34, 39], which provided a valuable resource for MAS to develop soybean varieties with high seed isoflavone content. Previously, two major QTL consistently affected isoflavone content across multiple environments were mapped on Gm05 (LG A1) and Gm08 (LG A2) by Gutierrez et al. [30] and Yang et al. [35], respectively. Here, we mapped three new QTL (qTIA2_1 located in Sct_067-Satt470; qGCA2_1 and qGTA2_1 located in Sat_040-Satt233) associated with individual and total isoflavone. The qGTA2_1 was detected in four environments and explained 3.51%-11.58% of the phenotypic variation. This QTL was near to the major locus identified by Yang et al. [35] that controlled the same trait GT, suggesting that qGTA2_1 might be an enzyme-related locus. The qGCA2_1 and qGTA2_1 were identified between the same marker interval of Sat_040-Satt233, implying that there could be some genetic factors regulating the accumulation of GC and GT. These stable QTL were responsible for large *a* effects (Table 2). As suggested by Tanksley [48], QTL with higher *a* effects are more likely to be stable across multiple environments. Most of the QTL discussed above with higher *a* effects (Significant at P = 0.01) were stable across at least three environments (Tables 1 and 2). These seven novel major QTL were selected to do the GGE Bioplot analysis, and only explaineding 59% of the G and G × E variation of seed isoflavone, which was lower than other’s studies [23, 31]. This could be caused by few QTL involved. The contribution of G and G × E to seed isoflavone phenotypic variation could be increased to 79% after the excluding of E4 (maybe a mega environment), indicating that E4 had significant influence on isoflavone content (S1 Fig.). Moreover, in order to examine the accuracy of the results by GGE Biplot analysis, QTLNetwork 2.0 software [49] was used to analyze the interaction between QTL and environments, and the result was similar to GGE Biplot results (S1 Table), indicating that QTL detected in multiple environments were more stable.

Among the newly identified QTL, the qTIF_1 shared the same marker Satt569 with qDZF_2, qGTF_1 and qTIF_2 in multi-environments, suggesting that there are some genetic elements, such as genes or factors could affect the accumulation of DZ, GT and TI (Table 1, Fig. 1). Additionally, six pairs of QTL overlapped with each other and shared the same marker interval, inferring that some genetic elements could regulate the accumulation of different isoflavone components in these intervals (Table 1). Among the 23 newly identified QTL, five QTL intervals were completely overlapped with our previously reported eQTL and a total of eleven candidate genes within the overlapped eQTL and QTL were identified [39]. For example, the newly identified QTL (qGCE_1, qGTE_1 and qTIE_1) located in the interval of Sat_124- Sat_380 shared the same marker Sat_380 with the eQTL qF3HE_1, implying the *F3H* gene in the phenylpropanoid pathway could affect the accumulation of GC, GT and TI.

In this study, many unstable QTL were detected in different environments (Table 1, Fig. 1), which were due to the weak expression of the QTL, QTL by environment interaction in the opposite direction to *a* effects, and/or epistasis (Tables 2 and 3). Therefore, the information of QTL by environment interaction should be considered if MAS was applied to the manipulation of quantitative traits. Since the 194 markers were not uniformly distributed, large gaps appeared with low marker density on chromosomes Gm02, 13 and 20, implying that more markers should be developed among these gaps and the authenticity of QTL should be further clarified.

The precise estimate of individual and total isoflavone content of soybean seed based on phenotype was difficult due to environment effect. Markers tightly linked to the QTL underlying isoflavone content would help to identify soybean lines containing higher isoflavone on the basis of genotype, to maximize the effectiveness of selection. Identification of stable QTL in multi-environments and fine mapping those loci could be desirable for identifying the underlying candidate genes or factors.

## Supporting Information

S1 Fig GGE Biplot analysis for the interaction of QTL and environment excluding E4. PC1: first principle component, PC2: second principle component.
(TIF)

S1 Table Additive × additive epistatic effect and their environmental interaction effect of QTL associated with individual and total isoflavone at RIL population using QTLNetwork 2.0 software. (DOCX)
