# Supplementary figures and images for: Mapping Isoflavone QTL with Main, Epistatic and QTL × Environment Effects in Recombinant Inbred Lines of Soybean
Source: PLoS One. 2015 Mar 4;10(3):e0118447. doi: 10.1371/journal.pone.0118447 (PMC4349890; doi:10.1371/journal.pone.0118447)

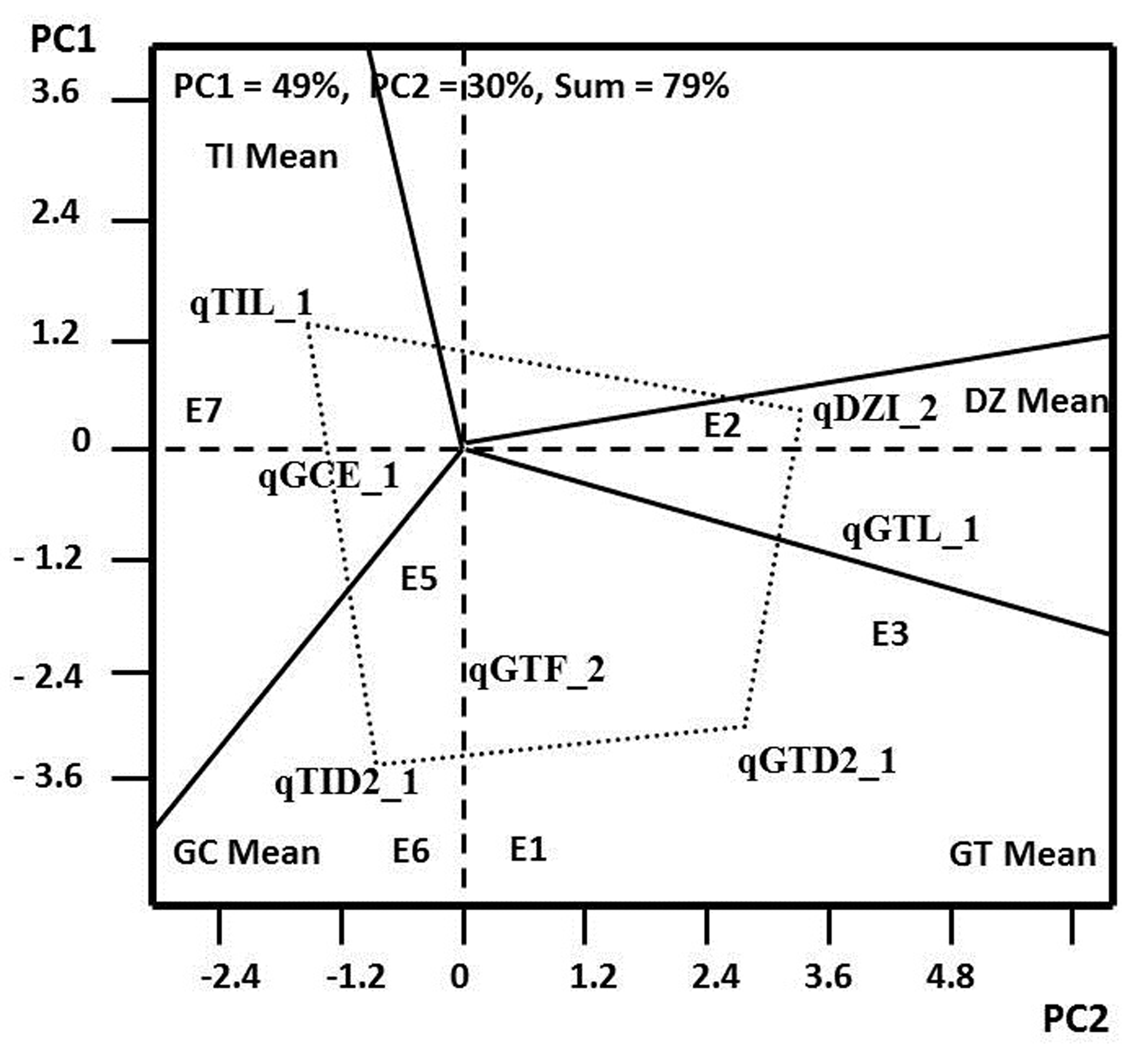

Supplement: S1 Fig — PC1: first principle component, PC2: second principle component. (TIF) [file pone.0118447.s001.tif]
