# Supplementary material for: Mapping Isoflavone QTL with Main, Epistatic and QTL × Environment Effects in Recombinant Inbred Lines of Soybean
Source: PLoS One. 2015 Mar 4;10(3):e0118447. doi: 10.1371/journal.pone.0118447 (PMC4349890; doi:10.1371/journal.pone.0118447)
Supplement: S1 Table — (DOCX) [file pone.0118447.s002.docx]

**S1 Table. Additive × additive epistatic effect and their environmental interaction effect of QTL associated with individual and total isoflavone at RIL population using QTLNetwork 2.0 software.**

| Traits | QTL_i | Marker interval_i | Site_i | QTL_j | Marker interval_j | Site_j | h^2^(aa)(%) | h^2^(aae)(%) | AA | AAE1 | AAE2 | AAE3 | AAE4 | AAE5 | AAE6 | AAE7 |
| --- | --- | --- | --- | --- | --- | --- | --- | --- | --- | --- | --- | --- | --- | --- | --- | --- |
| GC | qGCE_1 | Sat_124-Sat_308 | 16.3 | qGTL_1 | Sat_113-Sat_320 | 58.6 | 0.26 | 1.34 | -0.48** | 0.31** | -0.57** | 0.36** | 0.85** | -0.14** |  | -0.8 9* |
|  | qGCI_1 | Satt239-Satt330 | 33.1 | qTID2_1 | Sat_022-Satt208 | 21.4 | 0.51 | 2.41 | -0.13** | 0.23** |  | -0.56** |  | 0.32** | 0.61** |  |
|  | Satt144 | Satt144-Sat_074 | 27.8 | qDZI_2 | Satt330-Satt239 | 33.1 | 0.24 | 0.72 | 0.38** |  | 0.28** |  | -0.96* |  | -0.36* | -0.17** |
| GT | qGTD2_1 | Satt186-Satt226 | 50.9 | qGTL_1 | Sat_113-Sat_320 | 58.6 | 0.06 | 0.27 | -0.19* | 0.10** | -0.37* | 0.24** |  | -0.09* |  |  |
|  | qGTF_1 | Satt569-Satt144 | 13.5 | qGTF_2 | Satt149-Sat_234 | 41.2 | 2.77 | 2.29 | 0.14** | 0.13** | -0.24* | 0.16** | -0.21* | -0.03** | 0.01** | 0.02** |
| TI | qTIF_2 | Satt569-Satt144 | 13.4 | qGTF_2 | Satt149-Sat_234 | 41.2 | 0.88 | 1.80 | 0.27** | 0.09** | -0.04* | 0.18** | -0.06* | -0.36** | 0.02** |  |

*Significant at P = 0.05, **Significant at P = 0.01, respectively.

^a^ DZ: Daidzein; GC:Glycitein; GT: Genistein; TI: Total isoflavone

^b^ The nomenclature of the QTL included four parts : QTL, trait, linkage group name and QTL order in the linkage group, respectively.

^c^ *a*: additive effect;

^d^ *a*×E: additive × environment effect;

^e^ E1: at Harbin in 2005, E2: at Harbin in 2006, E3: at Hulan in 2006, E4:at Suihua in 2006, E5: at Harbin in 2007, E6: at Hulan in 2007, E7: at Suihua in 2007.
